# Common interacting genetic variation shapes susceptibility to type 1 diabetes in a Colombian Caribbean community: In search of shared genetic markers

**DOI:** 10.1016/j.gendis.2023.06.027

**Published:** 2023-08-02

**Authors:** Gloria Garavito-De Egea, Alex Domínguez-Vargas, Jorge I. Vélez, Gustavo Aroca, Luis Fang, Elkin Navarro-Quiroz, Zilac Espitaleta, Kenny Del Toro-Camargo, Leticia Martínez-Ariza, Tatiana González-Vargas, Susana García, Mauricio Arcos-Burgos, Eduardo Egea

**Affiliations:** a Universidad del Norte, División Ciencias de la Salud, Barranquilla, Colombia; b Universidad Simón Bolívar, Facultad de Ciencias de la Salud, Barranquilla, Colombia; c Clínica de la Costa, Grupo de Investigación en Nefrología, Barranquilla, Colombia; d Universidad de Antioquia, Grupo de Investigación en Psiquiatría (GIPSI), Departamento de Psiquiatría, Instituto de Investigaciones Médicas, Facultad de Medicina, Medellín, Colombia

Genome-wide association studies (GWASs) have identified hundreds of loci across the human genome conferring susceptibility to autoimmune diseases (AIDs), some of which are shared between more than two diseases. However, this univariate approach has limitations in detecting complex genotype-phenotype correlations. In this work, we carried out whole-exome sequencing of Colombian Caribbean patients with type 1 diabetes (T1D), lupus nephritis (LN), and juvenile idiopathic arthritis (JIA), to evaluate functional exomic variation, *i.e.*, single nucleotide polymorphisms (SNPs), and to outline common and rare variations underpinning the susceptibility to these autoimmune diseases. Single and multi-locus linear mixed-effects models fit the data to identify T1D-associated genomic variants and the most likely genetic architecture underpinning AID risk. Variations associated with T1D susceptibility pointed to genes related to glycoprotein oligosaccharide biosynthesis, phospholipid binding, pancreatic adenocarcinoma, systolic blood pressure, and fasting insulin metabolism, among others that highlight *MGAT5* (*P*_FDR_ = 1.64 × 10^−22^), *RUNX1* (*P*_FDR_ = 1.8 × 10^−12^), *PSD3* (*P*_FDR_ = 8.1 × 10^−12^), and *HLA-DBP2* (*P*_FDR_ = 2.18 × 10^−9^). Our study outlines oligogenic common variation underpinning the susceptibility to develop T1D. These genetic polymorphisms are also shared by patients with other AIDs such as LN and JIA, indicating that the shared genetic architecture (defined by pleiotropy and epistasis) shapes the genetic susceptibility of these disorders in this multiethnic population.

AIDs are estimated to impact 5%–8% of people living in America, representing a public health impact and a significant epidemiological burden. GWASs suggest that a set of shared genetic risk factors is underlying the etiology of AIDs.^1^ It is noteworthy that several AIDs show a clear family grouping, such as inflammatory bowel disease, while others, such as T1D, autoimmune thyroiditis, and celiac disease, can manifest as comorbid diseases among them.^2^ Indeed, the gene-sharing concept between AIDs is not clear to date, nor is it ruled out that this is due to ‘pleiotropic’ factors that predispose to multiple AIDs through shared mechanisms or if numerous independent risk factors are overlooked or add up to give these endotypes.^2^^,^^3^

Genetic evidence has stated that around 44% of SNPs found in GWASs on AIDs are shared by two or more of the following diseases: celiac disease, Crohn's disease, psoriasis, multiple sclerosis (MS), rheumatoid arthritis, T1D, and systemic lupus erythematosus. Moreover, T1D, LN, and JIA have previously been considered part of an AID cluster with genetic and clinical interactions.^1^ Several epidemiological studies, along with our findings, support overlapping genetic factors, for example, shared heritability in autoimmune diseases.^3^^,^^4^ In previous studies, our group demonstrated that transmission of AID susceptibility is fit either for i) the effects of major Mendelian loci or ii) oligogenic cooperating loci, or for iii) the inclusive models of hundreds of loci interacting with environmental effects.^5^

In this study, we report the analysis of whole-exome sequencing of 75 patients with autoimmune phenotypes (T1D, LN, and JIA, *n* = 25 from each group) ascertained from Barranquilla, the capital city of the Atlántico state in the Colombian Caribbean coast. We studied the association of common polymorphic variants (minor allele frequency ≥0.01) with T1D using single- and multi-locus linear mixed-effect models (SLMEM and MLMEM, respectively) with up to 10 steps in the backward/forward optimization algorithm.

The demographic characteristics of individuals included in this study are summarized in Table S1. The mean age for study participants was 20 ± 9.6 years. There were 55 (73%, 55/75) women and 20 men (27%, 20/75). Symptom lengths went from two to up to over 20 years. LN patients described a disease debut at 13.9 ± 2.6 years of age on average, T1D patients at 6.7 ± 4 years of age, and JIA patients reported a diagnosis of the disease at 6.5 ± 3.9 years of age.

DNA from patients was subject to whole exome capture, amplification, and sequencing. We identified a total of 2,779,380 SNPs. This number was reduced to 87,638 common variants with potential functional effects at the end of the filtering process. These variants were harbored in chromosomal regions reported to confer susceptibility to T1D and manually compiled and curated by our group. Manhattan plots depicting genome significances reached after the maximization of three MLMEMs (additive, dominant, and recessive) are presented in Figure 1.

**Figure 1:**
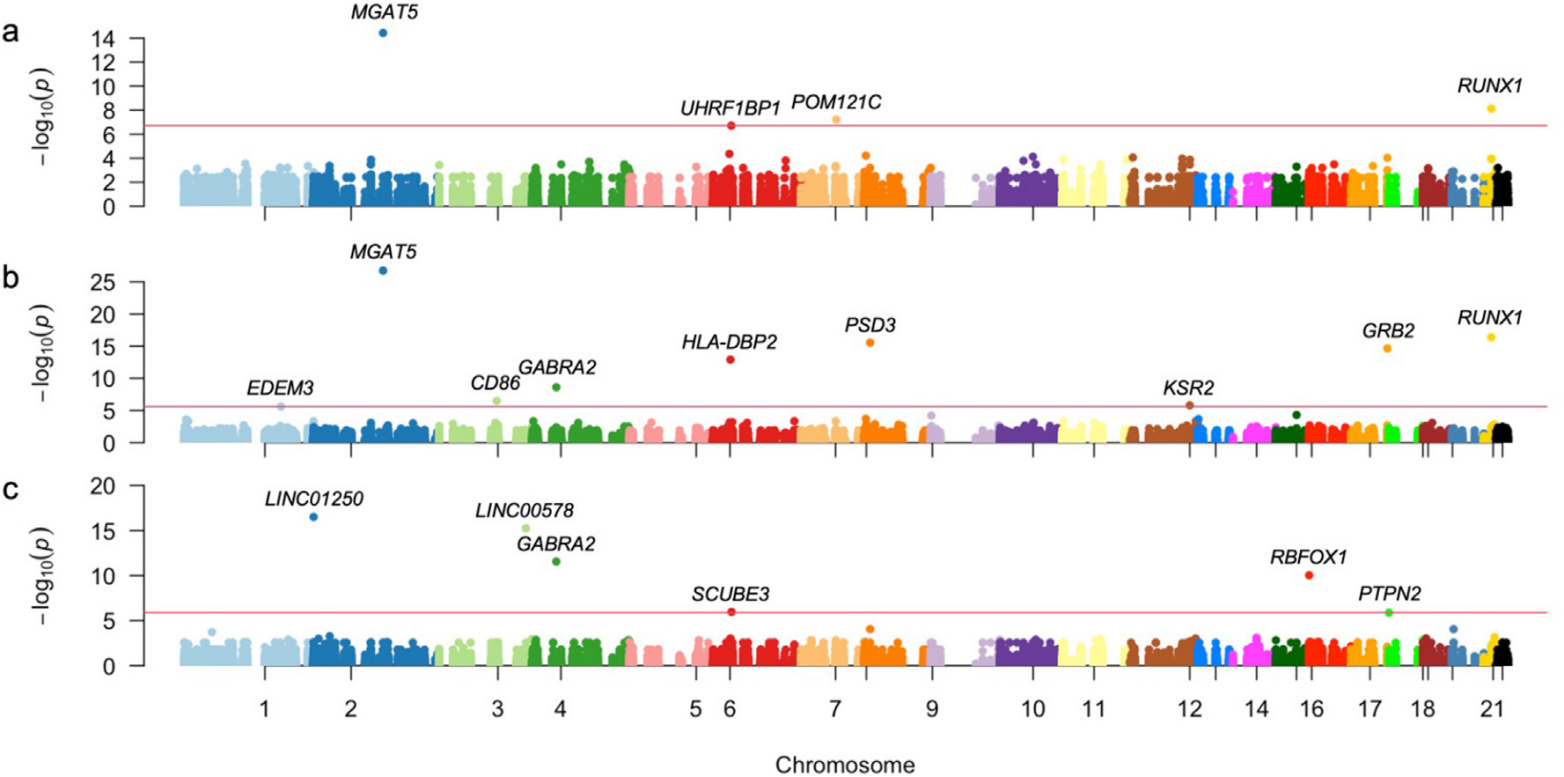
Genome-wide association analysis of patients (*n* = 75). The Manhattan plots of the association results are shown when **(a)** additive, **(b)** dominant, and **(c)** recessive multi-locus linear-mixed effects are used. The plots show the top markers detected with FDR-corrected *p-*values below 0.05 presenting –log_10_ (*P*) versus the SNPs coordinates throughout the genome. FDR, false discovery rate.

We contrasted several MLMEMs, including all 87,638 common variants in genes previously reported to confer susceptibility to T1D. An MLMEM with four, nine, and six steps in the forward/backward selection algorithm were selected under the additive (Table S2a and Fig. 1a), dominant (Table S2b and Fig. 1b), and recessive (Table S2c and Fig. 1c) forms of transmission, respectively. The advantage of these models is the inclusion of both fixed (genotype markers and covariates of any type) and random effects (family or population structure), the latter of which accounts for potential inbreeding by including a kinship matrix (the identity-by-descent matrix). In our case, the identity-by-descent matrix was estimated between all pairs of individuals using all markers located within genes of interest. A SLMEM assumes that all loci have a negligible effect on the trait (simulating a multifactorial model), while an MLMEM assumes that several interacting loci have a significant effect (non-linear epistatic effects).

Four variants confer susceptibly to T1D under the additive inheritance model (Table S2a). Of these, variants mapping to the *MGAT5* ($\hat{\beta }$ = 0.747, odds ratio/OR = 2.11, *P*_FDR_ = 3.18 × 10^−10^), *POM121C* ($\hat{\beta }$ = 0.419, *OR* = 1.520, *P*_FDR_ = 1.75 × 10^−3^), and *UHRF1BP1* ($\hat{\beta }$ = 0.684, *OR* = 1.982, *P*_FDR_ = 4.28 × 10^−3^) genes confer susceptibility to T1D, while the variant mapping to *RUNX1* ($\hat{\beta }$ = 0.747, *OR* = 0.586, *P*_FDR_ = 3.19 × 10^−4^) is protective (Table S2a).

We identified nine variants associated with T1D under the dominant inheritance model (Table S2b). Of these, variants harbored in the *MGAT5* ($\hat{\beta }$ = 1.336, *OR* = 3.8, *P*_FDR_ = 1.64 × 10^−22^), *PSD3* ($\hat{\beta }$ = 0.947, *OR* = 2.578, *P*_FDR_ = 1.8 × 10^−12^), *HLA-DBP2* ($\hat{\beta }$ = 0.607, *OR* = 1.835, *P*_FDR_ = 2.18 × 10^−9^), *CD86* ($\hat{\beta }$ = 0.381, *OR* = 1.464, *P*_FDR_ = 3.97 × 10^−3^), *KSR2* ($\hat{\beta }$ = 0.418, *OR* = 1.519, *P*_FDR_ = 1.17 × 10^−2^), and *EDEM3* ($\hat{\beta }$ = 0.162, *OR* = 1.176, *P*_FDR_ = 2.33 × 10^−2^) genes confer susceptibility to T1D. In contrast, variants within the *RUNX1* ($\hat{\beta }$ = −0.968, *OR* = 0.379, *P*_FDR_ = 1.8 × 10^−12^), *GRB2* ($\hat{\beta }$ = −0.808, *OR* = 0.446, *P*_FDR_ = 4.88 × 10^−11^), and *GABRA2* ($\hat{\beta }$ = −0.223, *OR* = 0.8, *P*_FDR_ = 3.49 × 10^−5^) genes are protective.

In the recessive transmission MLMEM model, we identified six variants associated with T1D (Table S2c and Fig. 1c). Among these, variants harbored in the LINC00578 ($\hat{\beta }$ = −1.833, *OR* = 0.159, *P*_FDR_ = 2.43 × 10^−11^) and *PTPN2* ($\hat{\beta }$ = −0.645, *OR* = 0.525, *P*_FDR_ = 1.87 × 10^−2^) genes are protective, and those within the *LINC1250* ($\hat{\beta }$ = 1.628, *OR* = 5.094, *P*_FDR_ = 3.49 × 10^−5^), *GABRA2* ($\hat{\beta }$ = 0.89, *OR* = 2.435, *P*_FDR_ = 3.49 × 10^−5^), *RBFOX1* ($\hat{\beta }$ = 0.825, *OR* = 2.282, *P*_FDR_ = 3.49 × 10^−5^), and *SCUBE3* ($\hat{\beta }$ = 0.724, *OR* = 2.062, *P*_FDR_ = 3.49 × 10^−5^) genes confer susceptibility to T1D.

In summary, our study outlines oligogenic common variation underpinning the susceptibility to develop T1D. These genetic polymorphisms are also shared by patients suffering from other diseases such as LN and JIA, indicating that the shared genetic architecture defined by pleiotropy and epistasis shapes the genetic susceptibility of these disorders in this multiethnic population. Given the predicted functional nature of these genetic variants, it is very likely that in this understudied multiethnic population, genes harboring these mutations are major contributors to AID immunopathology and provide new insights into the autoimmune tautology in this group of diseases.

## Ethics declaration

The study was conducted according to the guidelines of the Declaration of Helsinki and approved by the Ethics Committee of Universidad del Norte and Universidad Simón Bolívar, Barranquilla, Colombia (Approval # 00032, 13 October 2011). Informed consent was obtained from all subjects involved in the study.

## Author contributions

Conceptualization: G.G.-D.E., J.I.V., M.A.-B., E.E., and L.F. Methodology: G.G.-D.E., M.A-B., and J.I.V. Validation: J.I.V. and M.A.-B. Formal analysis: G.G.-D.E., J.I.V., M.A.-B., A.D.-V., and E.E. Data curation: G.G.-D.E., J.I.V., M.A.-B., S.G., K.T.-C., E.E., A.D.-V., T.G-V., and L.F. Visualization: J.I.V., K.T.-C., M.A.-B. and L.M.-A. Resources: G.G.-D., E.E., G.A., T.G-V., and Z.E. Writing—original draft preparation: G.G.-D.E, J.I.V., M.A.-B., E.E., and A.D.-V. Writing—review & editing: G.G.-D.E, J.I.V, A.D.-V, E.N.-Q., G.A, Z. E, S.G and E.E. Project administration: G.G.-D.E, J.I.V, and E.E. All authors have read and agreed to the published version of the manuscript.

## Conflict of interests

The authors declare no conflict of interests.

## Funding

This study was financed by Minciencias title proyect “Variantes genéticas y mutaciones genómicas identificadas por secuenciación de última generación (NGS) en familias colombianas: en busca de marcadores comunes de tautología autoinmune – No. 121577758377” and was partially supported from 10.13039/501100004245Universidad del Norte, Barranquilla, Colombia. The sponsor of the study had no role in study design, data collection, data analysis, data interpretation, or writing of the paper.
