# Supplementary material for: Common interacting genetic variation shapes susceptibility to type 1 diabetes in a Colombian Caribbean community: In search of shared genetic markers
Source: Genes Dis. 2023 Aug 2;11(4):101058. doi: 10.1016/j.gendis.2023.06.027 (PMC10951448; doi:10.1016/j.gendis.2023.06.027)
Supplement: Multimedia component 1 [file mmc1.docx]

Supplementary Materials for

**Common Interacting Genetic Variation Shapes Susceptibility to type 1 diabetes in a Colombian Caribbean Community. In Search of Shared Genetic Markers**

Gloria Garavito-De Egea^1, 2^, Jorge I. Vélez^1^, Alex Domínguez-Vargas^1^, Gustavo Aroca^1,2,3^, Luis Fang^1^, Elkin Navarro-Quiroz^2^, Zilac Espitaleta^3,2^, Kenny Del Toro-Camargo^1^, Leticia Martínez-Ariza^1^, Tatiana González-Vargas^3^, S. García^3^, Mauricio Arcos-Burgos^4^, Eduardo Egea^1,2#^

^1^Universidad del Norte, División Ciencias de la Salud, Barranquilla, Colombia.

^2^Universidad Simón Bolívar, Facultad de Ciencias de la Salud, Barranquilla, Colombia.

^3^Clínica de la Costa, Grupo de investigación en Nefrología, Barranquilla, Colombia.

^4^Universidad de Antioquia, grupo de Investigación en Psiquiatría (GIPSI), Departamento de| Psiquiatría, Instituto de Investigaciones Médicas, Facultad de Medicina, Medellín, Colombia.

^#^ Correspondence should be directed to Dr. Eduardo Egea, MD, Ph.D., Department of Medicine, Universidad del Norte, Barranquilla, Colombia. E-mail: [eegea@uninorte.edu.co](mailto:eegea@uninorte.edu.co).

**This file includes:**

Materials and Methods

Table S1 to S2

**Materials and Methods**

**Subjects**

We studied 75 non-family history-related individuals, 20 males (27%) and 55 females (73%), from the metropolitan area of Barranquilla, Colombia. A total of 25 individuals were diagnosed with T1D, 25 with LN, and 25 with JIA. All individuals participated voluntarily and written informed consent was obtained from them directly or from their parents (in the case of children <18 years old). The Ethics Committee approved the study of Universidad del Norte, Barranquilla, Colombia (approval # 00032, October 13, 2011).

**Clinical Evaluation**

Patients with T1D were diagnosed following the guidelines of the American Diabetes Association based on plasma glucose criteria, either the fasting plasma glucose (FPG) or the 2-h plasma glucose (2-h PG) value after a 75-g oral glucose tolerance test (oGTT) and/or the Hba1c criteria.

All the patients had an onset of diabetes before the age of 18 years and had autoimmune T1D, confirmed by the positivity of at least one T1D-specific autoantibody at the time of diagnosis. The LN and JIA diagnosis was made following the American College of Rheumatology (ACR) diagnostic criteria. Patients with polyautoimmunity and other immune-related diseases were excluded. All the patients were referred to the study by their medical specialty.

**Genetic, Statistical, and Bioinformatic Analyses**

*DNA Extraction and preparation.* Five milliliters (5 ml) of venous blood were collected from the forearm vein of each patient after informed written consent was obtained. Samples were stored at 4^o^C briefly before genomic DNA was obtained. Extracted DNA was stored at -80^o^C until analysis. DNA purity was checked using the Nanodrop (OD260/280 ratio). DNA degradation and contamination were monitored on 1% agarose gels. DNA concentration was measured using Qubit. Only DNA samples with OD260/280 ratio between 1.8~2.0 and concentration above 1.0 ug were used to prepare sequencing libraries.

*Library Preparation for Sequencing.* Agilent liquid phase hybridization was applied to efficiently enrich whole exons sequenced on an Illumina platform. Sequencing libraries and capture used Agilent SureSelect Human All ExonV5/V6 (Agilent Technologies, CA, USA) with reagents recommended by the instruction manual and following experimental procedures for optimal results.

*Next Generation Sequencing.* Genomic DNA was randomly fragmented to 180-280bp with a Covaris cracker, and then DNA fragments were ended polished, A-tailed, and ligated with the full-length adapter for Illumina sequencing. Fragments with specific indexes were hybridized with more than 543,872 biotin-labeled probes after pooling, then magnetic beads with streptavidin were used to capture 334,378 exons from 20,965 genes. Libraries were sequenced after PCR amplification and quality control.

*Bioinformatics Analysis.* All sequenced data were quality assessed (base quality distribution, nucleotide distribution, and presence of adapters, chimeras, and other contaminants) to identify/remove low-quality data/samples from further analysis. All high-quality data were mapped to the human genome assembly using the *bwa-mem* algorithm. Aligned files were processed using Genome Analysis Tool Kit (GATK) for base quality recalibration, indel realignments, and duplicate removal. This was followed by SNP and INDEL discovery and genotyping (plus phasing where applicable) following the GATK Best Practices recommendations. All variant calls were subject to variant quality score recalibration and filtering to remove low-quality variants.

*Quality Control, filtering, and classification of exonic variants.* Genetic data were imported to Golden Helix®'s SVS 8.9.1(Golden Helix, Inc, Bozeman, MT, USA), and quality control was performed, as recommended by several authors, using the following criteria: (*i*) fitting to Hardy-Weinberg equilibrium with *P*-values > 0.05/*m* (where *m* is the number of markers included for analysis); (*ii*) a minimum genotype call rate of 90%; (*iii*) and presence of two alleles, controlling for excess of heterozygosis by evaluating the departure from expected *f* (inbreeding-coefficient) values*, i.e.,* significant deviation toward negative values of the *f* coefficient. Markers not meeting any of these criteria were excluded from analyses. Genotype and allelic frequencies were estimated by maximum likelihood. Variants with a minor allele frequency (MAF) ≥ 0.01 were classified as common and rare otherwise. Exonic variants with potential functional effects were identified using the annotations in the database for nonsynonymous SNPs' functional predictions (dbNSFP, GRCh37/hg19 genome assembly). This filter uses SIFT, Provean, PolyPhen-2, Mutation Taster, Mutation Assessor, Gerp^++,^ and PhyloP to predict a variant's deleterious effect and is fully implemented in the SVS 8.3.3 Variant Classification module. Additionally, we looked for the presence of variants causing clinical disorders and annotated them according to the last ClinVar report.

*GWAS analysis.* For the association analyses, individuals diagnosed with T1D were labeled as "cases" (*n*=25), and those diagnosed with other AIDs (i.e., LN and JIA) were labeled as "controls" (*n*=50). The main advantage of this approach is that all individuals have the same genetic, cultural, and geographical background. Even though none of the subjects involved in this study were biologically related, which reduces potential selection biases, the potential presence of microdifferentiation among these "ad-hoc built" subpopulations populations, was evaluated using the fixation index, better known as the *F*_st_ statistic. The estimated *F*_st_ value was 0.00018 (95% confidence interval [CI] = -0.00016-0.00053), which suggests that the case and control populations were homogeneous.

SLMEM and MLMEM models were implemented in Golden Helix®'s SVS 8.8.3. The optimal model was selected using a comprehensive exploration of multiple criteria, including the Extended Bayes Information Criteria (*eBIC*)*,* the Modified Bayes Information Criteria (*mBIC*)*,* and the Multiple Posterior Probability of Association (*mPPA*). After the estimation process using the forward/backward algorithm is finished, the coefficients $\hat{\beta}_{1},\hat{\beta}_{2},\ldots,\hat{\beta}_{m}$ were extracted and a hypothesis test of the form *H*_0,_*_i_*:$\beta_{i}=0$ vs. *H*_1,_*_i_*:$\beta_{i}\neq0$ was performed for the *i*th genetic variant to obtain the corresponding *P*-value (*i*=1,2…,*m*). Thus, the series *P*_1_, *P*_2_,…,*P_m_* of *P*-values was subsequently corrected for multiple testing using the false discovery rate (FDR). Given that this research makes part of an exploratory enterprise, we model different forms of transmission and maximized models following additive, dominant, and recessive inheritance. In order to evaluate the potential effect of covariates in T1D susceptibility, we included age at diagnosis, age at assessment and gender as interacting factors.

**Table S1.** Demographic characteristics of individuals included in this study.

|  |  |  |  |  |  |
| --- | --- | --- | --- | --- | --- |
|  | **Parameter** | **General** | **SLE** | **T1D** | **JIA** |
|  |  | n: 75 | n: 25 | n: 25 | n: 25 |
| **Sex** |  |  |  |  |  |
|  | Male | 20 (27 %) | 4 (16 %) | 9 (36 %) | 7 (28 %) |
|  | Female | 55 (73%) | 21 (84 %) | 16 (64 %) | 18 (72 5) |
|  | Ratio (Female / Male) | 2.7 / 1 | 5.25 / 1 | 1.7 / 1 | 2.5 / 1 |
| **Age** |  |  |  |  |  |
|  | Children (6 - 11 years) | 13 (17.3 %) | 1 (4 %) | 2 (8 %) | 9 (36 %) |
|  | Adolescents (12 - 17 years) | 17 (22.6 %) | 5 (20 %) | 4 (16 %) | 8 (32 %) |
|  | Adults (>18 years) | 45 (60 %) | 19 (76 %) | 19 (76 %) | 8 (32 %) |
| **Diagnosis of the disease (years)** | |  |  |  |  |
|  | Mean ± SD | 9.05 ± 4.6 | 13.9 ±2.6 | 6.72 ± 4.03 | 6.52 ± 3.96 |
|  | 95% CI of Mean | 7.9 - 10.2 | 12.8 - 15 | 5.05 - 8.39 | 4.88 - 8.16 |
| *T1D: Type 1 Diabetes. SLE: Systemic Lupus Erythematosus. JIA: Juvenile Idiopathic Arthritis. SD: Standard deviation. CI: Confidence interval.* | | | | | |

**Table S2.** Results of the association analysis via multi-locus LMEMs under the **(a)** additive, **(b)** dominant, and **(c)** recessive models of inheritance for 75 patients with AIDs from Barranquilla, Colombia.

| **(a)** | |  |  |  | |  |  | |  | | |  |  | |
| --- | --- | --- | --- | --- | --- | --- | --- | --- | --- | --- | --- | --- | --- | --- |
| **Chr** | **Position*^a^*** | **Gene*^b^*** | **Ref/Alt** | **MAF** | ***P*** | | | $\hat{\boldsymbol{\beta}}$ | | **SE(**$\hat{\boldsymbol{\beta}}$**)** | ***P*_FDR_** | | |  |
| 2 | 135,005,748 | *MGAT5* | G/A | 0.047 | 3.64E-15 | | | 0.747 | | 0.074 | 3.19E-10 | | |  |
| 21 | 36,287,713 | *RUNX1* | A/G | 0.040 | 7.27E-09 | | | -0.534 | | 0.081 | 3.19E-04 | | |  |
| 7 | 75,091,664 | *POM121C* | A/G | 0.040 | 6.00E-08 | | | 0.419 | | 0.069 | 1.75E-03 | | |  |
| 6 | 34,819,311 | *UHRF1BP1* | C/A | 0.027 | 1.95E-07 | | | 0.684 | | 0.118 | 4.28E-03 | | |  |
| **(b)** | |  |  |  | |  |  | |  | | |  |  | |
| **Chr** | **Position*^a^*** | **Gene*^b^*** | **Ref/Alt** | **MAF** | ***P*** | | | $\hat{\boldsymbol{\beta}}$ | | **SE(**$\hat{\boldsymbol{\beta}}$**)** | ***P*_FDR_** | | |  |
| 2 | 135,005,748 | *MGAT5* | G/A | 0.047 | 1.87E-27 | | | 1.336 | | 0.071 | 1.64E-22 | | |  |
| 21 | 36,287,713 | *RUNX1* | A/G | 0.040 | 4.10E-17 | | | -0.968 | | 0.084 | 1.80E-12 | | |  |
| 8 | 18,804,396 | *PSD3* | G/A | 0.013 | 2.77E-16 | | | 0.947 | | 0.086 | 8.10E-12 | | |  |
| 17 | 73,404,906 | *GRB2* | T/C | 0.053 | 2.23E-15 | | | -0.808 | | 0.077 | 4.88E-11 | | |  |
| 6 | 33,086,686 | *HLA-DBP2* | C/T | 0.027 | 1.24E-13 | | | 0.607 | | 0.064 | 2.18E-09 | | |  |
| 4 | 46,388,848 | *GABRA2* | C/G | 0.213 | 2.39E-09 | | | -0.223 | | 0.032 | 3.49E-05 | | |  |
| 3 | 121,807,260 | *CD86* | C/T | 0.047 | 3.17E-07 | | | 0.381 | | 0.067 | 3.97E-03 | | |  |
| 12 | 118,002,999 | *KSR2* | T/C | 0.040 | 1.56E-06 | | | 0.418 | | 0.079 | 1.71E-02 | | |  |
| 1 | 184,693,047 | *EDEM3* | C/T | 0.340 | 2.39E-06 | | | 0.162 | | 0.031 | 2.33E-02 | | |  |
| **(c)** | |  |  |  | |  |  | |  | | |  |  | |
| **Chr** | **Position*^a^*** | **Gene*^b^*** | **Ref/Alt** | **MAF** | ***P*** | | | $\hat{\boldsymbol{\beta}}$ | | **SE(**$\hat{\boldsymbol{\beta}}$**)** | ***P*_FDR_** | | |  |
| 2 | 3,023,643 | *LINC01250* | T/C | 0.033 | 3.14E-17 | | | 1.628 | | 0.142 | 2.76E-12 | | |  |
| 3 | 177,172,699 | *LINC00578* | A/G | 0.040 | 5.54E-16 | | | -1.833 | | 0.171 | 2.43E-11 | | |  |
| 4 | 46,262,822 | *GABRA2* | A/G | 0.053 | 2.67E-12 | | | 0.890 | | 0.104 | 7.81E-08 | | |  |
| 16 | 6,446,332 | *RBFOX1* | T/G | 0.060 | 9.11E-11 | | | 0.825 | | 0.107 | 2.00E-06 | | |  |
| 6 | 35,208,770 | *SCUBE3* | C/T | 0.027 | 1.05E-06 | | | 0.724 | | 0.134 | 1.85E-02 | | |  |
| 18 | 12,823,403 | *PTPN2* | T/G | 0.040 | 1.28E-06 | | | -0.645 | | 0.121 | 1.87E-02 | | |  |

*^a^* UCSC GRCh37/hg19 coordinates.

*^b^*Nearest gene.

Chr: Chromosome, Ref/Alt: Reference and Alternate Allele, MAF: Minor Allele Frequency, *P*: *P-*value, $\hat{\beta}$: Estimated regression coefficient in the single- and multi-locus linear mixed effects model, SE($\hat{\beta}$): Standard error (SE) of $\hat{\beta}$, *P*_FDR_: FDR-corrected *P-*value, FDR: False Discovery Rate.
